# Feeding Practices, Parent Perceptions, and Diet Diversity in a Sample of Children Aged 0–5 Years from Western Sydney, Australia: A Mixed Methods Study

**DOI:** 10.3390/nu16020198

**Published:** 2024-01-08

**Authors:** Anjana Iyer, Katherine Kent, Kaitlyn Brunacci, Kingsley Emwinyore Agho, Catharine A. K. Fleming

**Affiliations:** 1 School of Science, Western Sydney University, Locked Bag 1797, Penrith, NSW 2751, Australia; 2 School of Medicine, Western Sydney University, Campbelltown, NSW 2560, Australia; 3 School of Health Sciences, Western Sydney University, Campbelltown, NSW 2560, Australia; 4 School of Medical, Indigenous and Health Sciences, Faculty of Science, Medicine and Health, University of Wollongong, Wollongong, NSW 2522, Australia

**Keywords:** child, diet, feeding behaviour, breastfeeding, nutritional requirements, diet diversity

## Abstract

(1) Background: Sub-optimal feeding practices and dietary intakes in childhood are associated with poor health outcomes in adulthood. This mixed methods study aims to profile feeding practices, parent perceptions, and dietary diversity in a sample of children aged 0–5 years (*n* = 29) from Western Sydney, Australia. (2) Methods: In 2019, semi-structured interviews were conducted with parents, exploring their child’s feeding practices. An online quantitative survey was used to assess children’s feeding history. Nutrient intakes and dietary diversity scores were assessed using an online 24-h dietary recall. Quantitative data were analysed using descriptive statistics and one-way ANOVA, while qualitative data were analysed using inductive thematic analysis. (3) Results: The analysis showed that 27.6% of children were exclusively breastfed until 6 months of age and that 62.1% of children were introduced to solids before 6 months. Over 60% of children achieved minimum dietary diversity. The thematic analysis identified four themes, including mothers’ feeding choices, mothers’ perceptions of their children’s diet, influences on feeding choices, and mothers’ personal experiences. (4) Conclusions: The feeding history of over half the children in this sample indicated non-compliance with Australian and WHO infant feeding guidelines. The thematic analysis revealed various possible influences on parent feeding choices that can be used to inform targeted support.

## 1. Introduction

Early nutrition can influence the later development of obesity and other risk factors for non-communicable diseases [1]. The first 1000 days of life from conception through to 24 months of age are particularly crucial for a child’s growth and development [2], and childhood dietary habits can influence eating practices in adulthood [3]. In Australia, 25% of children aged 2–17 years are either overweight or obese [4], and the prevalence of obesity is especially high among children from culturally and linguistically diverse backgrounds [5].

Various dietary guidelines exist that aim to optimise health outcomes in children. The Australian Infant Feeding Guidelines (AIFGs) [6] encourage mothers to exclusively breastfeed children from birth to six months of age. They also recommend introducing solid foods from six months onwards and continuing breastfeeding until 12 months and beyond. For children over 12 months of age, the Australian Dietary Guidelines (ADGs) [7] recommend consuming a diverse range of foods from the five core food groups. Despite these guidelines, the Australian Institute of Health and Welfare [8] recently reported that most Australian children aged 2–3 years were meeting the recommended intakes of fruit and dairy but not vegetables, meats, and grains. Children in this age group also consume large amounts of added sugars and saturated fats via excess discretionary foods [9]. The use of pureed baby foods in commercial squeeze pouches is also a potential source of excess energy [10]. A recent national study [11] found sub-optimal intakes of core food groups and widespread intakes of discretionary foods among children aged 1–2 years. However, beyond this, there is limited recent research on young children’s diets in focussed regions of Australia, including Western Sydney, thereby limiting local intervention.

Western Sydney is an ethnically diverse region; the 2016 Australian census indicated that 49.8% of its population was overseas-born, with India and China being the main countries of birth [12]. Investigation into children’s diets in this region is important as dietary habits and food preferences may differ from the national average. Understanding the unique dietary patterns in this population may facilitate the development of specific interventions that reflect the diverse cultural, dietary, and socioeconomic factors influencing children’s diets. The present study therefore aims to achieve the following:

(a) Describe the feeding history (including breastfeeding and squeeze pouch use), diet diversity, and nutrient intake in a sample of children aged 0–5 years from Western Sydney.
(b) Explore the factors underlying decisions around feeding practices made by parents for the children in this sample.

## 2. Materials and Methods

### 2.1. Participants

Parent–child dyads (*n* = 29) were recruited for this cross-sectional, mixed methods study via community groups in the Western Sydney area. Parents were eligible for recruitment if they had a child aged 0–5 years who spoke English and lived in Western Sydney. Ethical approval was provided by the Western Sydney University Human Research Ethics Committee (H13055) on 21 February 2019, and written informed consent was obtained from each parent prior to participation.

### 2.2. Study Design and Data Collection

#### 2.2.1. Design

A mixed methods approach using one-on-one, in-depth, semi-structured interviews and an online questionnaire was used to investigate dietary intake, feeding behaviours, and factors that influence parent decisions around how they feed their children aged 0 to 5 years. Participants were recruited via social media and participant snowballing. Participants were given an information sheet, and consent was obtained before the interview. Participants were informed of their confidentiality and voluntary participation in this study.

#### 2.2.2. Qualitative Data Collection

One-on-one, semi-structured interviews were completed with parents and designed to be conversational in style. All interviews were conducted via the phone by research officer KB, digitally recorded and transcribed verbatim. The interview guide included open-ended questions around feeding practices, the use of commercial food pouches, and decisions around their child’s feeding journey. Prompts were used by the interviewer for clarification and to encourage the exploration of ideas.

#### 2.2.3. Quantitative Data Collection

A self-administered online questionnaire, completed by the parents, collected each parent and child’s age, sex, Indigenous status, length/height, and weight. Each parent’s ethnicity, education level, and suburb of residence were also collected. Body mass index (BMI) was thereafter calculated according to weight in kg/[length/height in m]^2^. The children’s BMIs were standardised for age and sex, using Epi Info™ (version 7.1.5) [13], to give BMI *z*-scores. Each mother–child dyad’s SES was determined by locating their home suburb on the Australian Bureau of Statistics’ Index of Relative Socio-economic Advantage and Disadvantage (IRSAD) [14]. This index scores Australian suburbs to indicate their socioeconomic standing and groups’ scores into deciles, where higher deciles correspond to higher levels of socioeconomic advantage.

A second self-administered online questionnaire, adapted from the 2010 Australian National Infant Feeding Survey [15], surveyed the mothers about their child’s feeding history, including first feed, breastfeeding duration, age at introduction to solid foods, and use of squeeze pouches.

Comprehensive dietary intake data were collected using the Automated Self-Administered 24-h Dietary Assessment Tool (ASA24, National Cancer Institute, Bethesda, MD, USA) [16], which allowed the parent to complete a one-day dietary recall for their child. From the recall data, diet diversity scores (DDSs) were calculated for each child according to the World Health Organization’s minimum dietary diversity (MDD) measure [17]. DDSs were determined as follows: a score of 1 was given for each food group consumed out of eight groups (1. breastmilk; 2. grains, roots, tubers, and plantains; 3. pulses, nuts, and seeds; 4. dairy products; 5. flesh foods; 6. eggs; 7. vitamin A-rich fruits and vegetables; and 8. other fruits and vegetables) to give a total possible score of 8. A full score of 8 indicates optimal diet diversity, whilst minimum diet diversity is defined by a score of 5 [17].

To calculate macro- and micro-nutrient intakes, recall data were entered into FoodWorks 9 (v9.0, Xyris Pty Ltd., Brisbane, Australia, 2017). Each child’s nutrient adequacy, based on their age, was ascertained by cross-checking their nutrient intakes with the estimated average requirements (EARs) (or adequate intakes (AIs) when the EARs were not available) provided in the nutrient reference values (NRVs) for Australia and New Zealand [18]. The NRVs were also used to determine each child’s estimated energy requirement (EER) based on their age and sex. Acceptable macronutrient distribution ranges (AMDRs) based on the children’s EERs were used to determine the adequacy of macronutrients for which EARs or AIs do not exist. For example, since the NRVs only provide AIs for total fats and carbohydrates for children aged 12 months or below, the AMDRs for these nutrients were used to assess the intakes of older children.

### 2.3. Analysis

A sub-analysis was conducted on the qualitative and quantitative data using a mixed methods design, incorporating thematic analysis and statistical analyses, respectively.

#### 2.3.1. Statistical Analysis

Analyses were conducted at the mother–child dyad level. Depending on their normality, as assessed using the Shapiro–Wilk test, continuous data were summarised using descriptive statistics, such as mean and standard deviation (SD), median, and interquartile range (IQR). Categorical data were summarised as counts (*n*) and percentages of each category. One-way analysis of variance (ANOVA) was conducted to compare the means of more than two variables, and cross-tabulations with χ^2^ statistics were used to determine any relationships between categorical variables. The significance level for all analyses was set at *p* < 0.05. All analyses were conducted using SPSS version 28 (IBM Corp., Armonk, NY, USA).

#### 2.3.2. Thematic Analysis

Thematic analysis [19] was guided by a social constructionist framework to acknowledge the nature of individual realities that are constructed through language and a broad range of social discourses [20]. The data gathered were initially read and re-read to increase familiarisation by author AI. From this, initial codes were extracted as in vivo codes or descriptive codes. The initial codes were then transcribed and collated for review and consensus by CF and AI. During this process of review, repeating codes were collapsed, and similar codes were grouped together under the most appropriate name. A coding framework was then developed consisting of higher-order codes and sub-codes according to this study’s aim. The coding framework was confirmed and verified through double coding of the first interview by CF and AI. With the confirmation and consensus of the coding framework, all transcripts were coded using QSR NVivo (release 1.7.1) qualitative coding software. Through the coding process, summary themes were developed and synthesised. This coding process ensured that the themes accurately reflected participants’ experiences. The final themes were then refined and named, and text extracts capturing participants’ experiences were selected to illustrate the results.

## 3. Results

### 3.1. Quantitative

#### 3.1.1. Participants

Both demographic and anthropometric characteristics of the mother–child dyads are summarised in Table 1. Most children (*n* = 26, 89.7%) were aged over 12 months.

#### 3.1.2. Feeding History

Data on breastfeeding history, age at introduction to solids, and squeeze pouch use are provided in Table 2. Half of the six children who were still being breastfed were between 6–12 months of age, whilst the remaining children were breastfed until 1, 2, or 5 years of age. The percentage of ever-breastfed children was 93.1% (*n* = 27), and just over a quarter of children (*n* = 8, 27.6%) were exclusively breastfed to 6 months of age. Of the children aged over 12 months, 53.8% (*n* = 14) had received any breastmilk beyond 12 months of age. The median age at introduction to solids was 5 (4–6) months. Out of the 24 children who had previously consumed food from a squeeze pouch, 29.2% (*n* = 7) of children used squeeze pouches at least once daily; 25% (*n* = 6) used them at least once weekly; and 16.7% (*n* = 4) used them at least twice monthly.

#### 3.1.3. Diet Diversity Score

**Figure 1:**
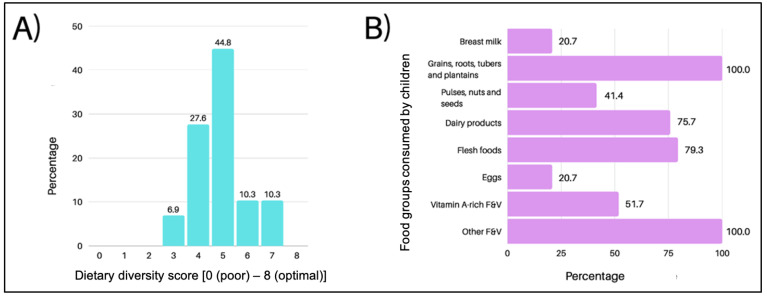
(**A**) Diet diversity scores of children; and (**B**) percentage of sample who consumed each food group.

Figure 1A displays the children’s DDSs. The median DDS was five (3–7), and most children (*n* = 19, 65.5%) met the MDD score. The percentage consumption of each food group is shown in Figure 1B.

#### 3.1.4. Nutrient Intakes

**Figure 2:**
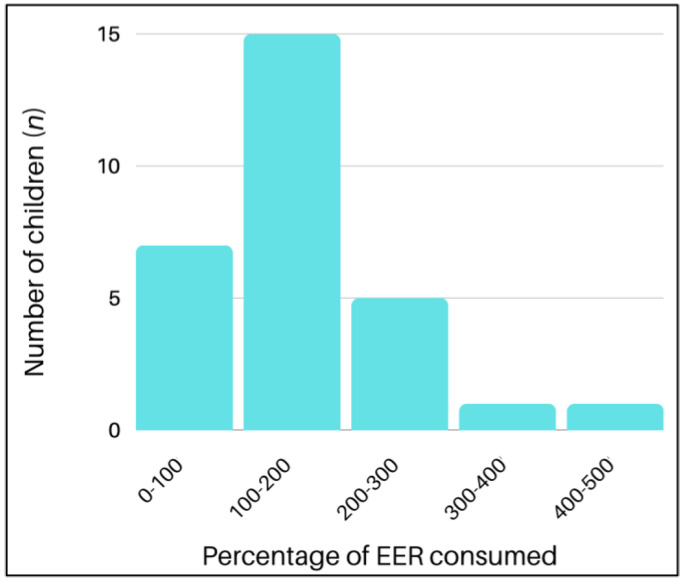
Percentage of estimated energy requirement (EER) consumed by children.

Table 3 presents the children’s energy and nutrient intakes and their nutrient adequacy. Figure 2 shows how many children met and/or exceeded their EERs and by what percentage. Most children (*n* = 22, 75.9%) met their EERs, and over half (*n* = 15, 51.7%) consumed between 100% and 200% of their EERs. A few children (*n* = 5, 17.2%) consumed 200% to 300% of their EERs, while one child (3.4%) each consumed over 400% and over 500% of their EERs, respectively.

While the upper limits for protein intake are not provided in the NRV, the upper bound of the AMDR for proteins (25% of energy) is recommended as an upper intake limit. Most children (*n* = 25, 86.2%) exceeded this threshold, having consumed over 25% of their EER as energy from proteins. No child below 12 months of age met their AI for total fat. Out of the children aged over 12 months, one child (3.8%) was below the AMDR for total fat (20–35% of EER), and 18 children (69.2%) exceeded the AMDR. Energy from saturated fats exceeded 10% of EERs for 82.8% (*n* = 24) of the full sample. Only two children under 12 months met their AI for carbohydrates. Of the children aged over 12 months, 53.8% (*n* = 14) exceeded the AMDR for this macronutrient (45–65% of EER). Over 50% of children met the EARs or AIs for all the measured micronutrients. Several children (*n* = 11, 37.9%) exceeded the upper level of zinc intake for their age, and one child (3.4%) exceeded the upper level of iron intake. Of the children aged over 12 months, for whom upper levels of sodium intake exist, 76.9% (*n* = 20) exceeded the upper level for their age. The use of squeeze pouches was associated with meeting protein and sodium requirements (both *p* = 0.026), and an association between squeeze pouch use and exceedance of sodium intake limits trended towards significance (*p* = 0.053).

### 3.2. Qualitative Analysis

The qualitative analysis of mothers’ responses indicated significant variation in ideals and beliefs surrounding their children’s dietary intake and decisions around feeding practices. The following analysis thematically examines the ways in which mothers from a sub-sample of Western Sydney experience and construct feeding decisions.

#### 3.2.1. Mothers’ Perceptions of Their Children’s Diet

A key theme that emerged and underpinned how and what a child was fed related to how a mother perceived their children’s dietary intake. Quotations are provided in Table 4. Many mothers perceived their children’s diet to be healthy in that they had limited ‘junk food’ intake, mostly ate fresh, home-cooked foods, or had large appetites. Conversely, some mothers noted that their children’s diets could be improved, with concerns being expressed about diet variety, picky eating, and whether their children were eating enough. When it came to feeding practices, a common sub-theme was mothers’ perceptions of their child’s readiness to eat solids, with several mothers noting that their child was showing interest in food, and some noting that their child was tolerating solid foods well.

#### 3.2.2. External Influences

Mothers discussed various external influences on their feeding choices, such as when to introduce solid foods to their child (Table 5). Common influences were family and friends and information from social media, books, and the internet, including guidelines. Some mothers mentioned information from mother’s groups and other mothers as an influence. Many mothers noted that they had limited to no discussions with healthcare providers about their child’s feeding. Some mothers noted a lack of consistency in information across various sources.

#### 3.2.3. Personal Experiences

A mother’s own personal experience was often discussed in relation to how they then chose to feed their child (Table 6). The sub-themes that were discussed included the mothers’ upbringing, experiences with older children, and experiences with illness or intolerance. Regarding upbringing, mothers discussed how their parents raised them and the influence of their culture. Some mothers discussed repeating feeding choices they made with their older children. Interestingly, several mothers also discussed how conditions such as eczema or dietary intolerances in their family impacted their feeding decisions for their child.

#### 3.2.4. Feeding Choices and Behaviours

Table 7 provides quotations regarding mothers’ feeding choices and behaviours. Mothers discussed foods they commonly chose to feed their child, including family foods, pureed or softened vegetables and fruits, and cereal foods. Regarding feeding behaviours, a few mothers discussed wanting their children to finish part or all of their food. Most mothers indicated that while they would not give their child full autonomy, they would allow their child to have some choice in the type and number of foods they eat. A common behaviour among mothers included providing a range of foods and letting their child choose from them. Some mothers discussed being guided by satiety cues from their children. Only two mothers discussed using food bribery.

## 4. Discussion

The present study aimed to describe the feeding history, diet diversity, and nutrient intake of a sample of children aged 0–5 years in Western Sydney, Australia, along with parents’ perceived drivers and perceptions of their children’s dietary intake. In this study, a limited number of infant feeding practices were in line with the AIFG; most children were not exclusively breastfed to 6 months, and very few received any breastmilk beyond 12 months. These findings parallel those of a recent study from Western Sydney [21], which indicated that 13.5% of children were exclusively breastfed at 6 months, and 25.5% were breastfeeding at 12 months. Similarly to the present study, these children were of a median age of 22 weeks when introduced to solid foods [22]. The present study also found that most children have consumed food from squeeze pouches, which can contain high levels of added sugars [23], going against the AIFG recommendation of “limiting intake of all foods with added sugars”. Such findings highlight the extent of non-compliance with the AIFG in Western Sydney and signal a possible need for region-specific interventions to improve infant feeding practices.

A larger proportion of children in the present study achieved minimum diet diversity, compared to another study by Leonard et al. [24], in which 29.5% of children from various northern Australian states met this standard. While grains and flesh foods (meat) were among the most commonly eaten food groups in both studies, fruits and vegetables were more widely consumed in the present study. These differences may be attributable to factors such as affordability, considering that Leonard et al. found that significantly more children consumed fruits during ‘pay week’. Vitamin A-rich fruits and vegetables, however, were consumed by only half of the sample in the present study. A 2008 study on children’s dietary intakes in Western Sydney [25] found that 6.8% of children were not meeting the EARs for vitamin A. The poor intake of vitamin A-rich fruits and vegetables observed in the present study suggests that an insufficient vitamin A intake may be a lingering issue among children in Western Sydney. However, further research is required to ascertain whether children’s vitamin A intakes in Western Sydney remain poor, since vitamin A could be derived from other sources. Intakes of legumes and eggs were also poor in the present study. Polak et al. [26] recommended cooking legumes in bulk and adding them as a side dish to meals to increase their intake. It is recommended that further research should incorporate longer dietary recalls or food frequency questionnaires to capture habitual dietary intake in Western Sydney to more accurately gauge the prevalence of such dietary patterns.

Several children in the current study reported large energy intakes, and most children’s macronutrient and sodium intakes exceeded the recommended limits, suggesting an intake of energy-dense foods or overconsumption tendencies within this sample. The ADG [7] recommends that intakes of saturated and trans fats together contribute no more than 10% of total energy; yet, most children’s intakes of saturated fat alone exceeded this threshold. The combination of these factors suggest excessive intakes of discretionary foods among this sample [9], which are also recommended by the ADGs to be consumed “only sometimes and in small amounts’’ for children from 2 years of age [7]. Consuming discretionary foods in moderation, or swapping them with healthier food options, may help reduce energy, sodium, and saturated fat intakes in children [27]. A study conducted in 2006 [28] observed high intakes of energy-dense, nutrient-poor foods among children in Western Sydney; the trends of the present study imply that children’s diets in the area have not improved since, which is of concern for long-term health outcomes.

Squeeze pouches were reportedly used by some children. Many commercially available children’s foods such as these have been identified as large contributors to intakes of energy, macronutrients, and sodium [10]. A recent study demonstrating the poor nutrient value and high energy content of squeeze pouch products [23] is supported by our findings of squeeze pouch use being associated with meeting protein and sodium requirements and potentially being associated with excessive sodium intake. The consumption of squeeze pouches may therefore account, at least in part, for the dietary intakes observed in this study. The frequency of squeeze pouch uses beyond this study sample, including the demographic predictors of use and impact on nutritional outcomes, remains an avenue for future research.

A study investigating dietary intakes within a nationally representative sample of Australian children aged 4–8 years [29] observed similar results to the present study; the majority of children met the recommendations for most micronutrients, although their dietary fibre intakes were low. The present study therefore indicates that low fibre intakes may be prevalent among Australian children of a wider age group. Strategies to increase fibre intake include eating a diverse range of plant-based foods and consuming foods fortified with fibre [30]. Several children in the current study exceeded the upper intake limits for zinc. A study from the USA [31] found that large proportions of a representative sample of American children exceeded the intake limits for zinc, primarily deriving this nutrient from food sources. Since most children in the current study consumed foods from several food groups during the recall period, the high intakes of zinc, and perhaps other nutrients, may thus be due to an excessive consumption of core foods.

The thematic analysis provided further insights into the feeding practices adopted by the mothers and the various influences on their feeding decisions. Given that Western Sydney is a multicultural region, and that cultural factors can influence feeding decisions [32], the mothers’ perceptions of their children’s diet may explain the deviation from the guidelines observed in this study. Some mothers reported feeding their children solids according to cues from their child that indicated interest in food; this may have prompted mothers to introduce solids earlier than recommended. Similar findings have been observed previously [33]. Personal experiences of the mothers, such as their upbringing, were also noted to bear influence on feeding decisions. The influence of parental upbringing on feeding practices was also observed in another Australian study [34], with parents continuing their family traditions of eating as a family and eating healthy.

Further, the observed energy and nutrient intakes may stem from behaviours that some mothers misperceived as healthy, including large appetites and high intakes of core foods such as fruits. Overestimation of children’s diet quality by mothers has been observed previously, and this may prevent timely interventions and perpetuate poor diet quality in the long term [35]. Some mothers were aware that their children’s diet could be improved. Given the sub-optimal feeding patterns observed in this study, future research could further explore barriers to healthy feeding among culturally diverse mothers in this region. Potential barriers found in this study, and possible avenues for intervention, may include inconsistent information sourced from relatives, the media, and the mothers’ own upbringings, in addition to a lack of communication from healthcare providers. Similar to the present study, Duncanson et al. [36] found social influences and the internet to be major sources of nutritional knowledge for mothers. The influence of other family members, such as grandparents, on feeding choices has also been documented [37]. A recent study [38] found that parents used social media to access health information and for social support. Therefore, a greater understanding of these motivators could allow for more targeted interventions to address the aforementioned barriers to healthy feeding choices.

Foods that were commonly discussed by mothers were core foods and foods eaten by the family. Some mothers perceived their children’s diets to be mostly healthy and that their discretionary food intake was occasional; yet, children in the current sample were found to have energy and sodium intakes that exceeded the recommended limits. This may be attributable to an excessive consumption of perceived healthier foods, such as commercially available baby and snack foods. This was also noted by Moumin et al. [11], who found that energy from discretionary foods constituted a median 10% of the daily energy intake in a sample of 475 Australian toddlers aged 12–24 months. Moumin et al. also noted that commercial toddler foods may be driving poor dietary intakes and increasing the risk of displacement of other food groups in the diet. Parents in the present study identified feeding behaviours such as pressure to eat and food bribery to a lesser extent, which may be contributors to possible overeating tendencies. The evidence has also shown restrictive behaviours to be associated with overeating prohibited foods, such as discretionary foods [39]. Alternatively, intakes of discretionary foods may have been underreported and healthy eating patterns exaggerated due to social desirability bias.

This study has a number of strengths, including the mixed methods design that was employed to gain a deeper understanding of the context of dietary patterns among children. Among the limitations of the present study is the determination of dietary diversity and adequacy from one day of dietary recall data, which may not reflect a child’s regular eating patterns. Physical activity levels and individual metabolic differences were also not considered when determining the EERs. Since the AMDRs were calculated from these EERs, a degree of uncertainty would be associated with the children’s nutrient adequacy with respect to being within or outside the recommended macronutrient intake ranges. The mothers’ knowledge about nutrition and feeding guidelines was also not assessed. Confounders were not factored into this study’s analyses due to limitations with the study sample size. The small sample size also limits the generalisability of this study’s findings beyond the study sample. Due to this small sample, differences in nutrient intake between ethnic and other demographic groups could not be investigated. To extend this preliminary research, further studies employing adequately powered samples are needed.

## 5. Conclusions

Feeding practices at variance with guidelines and sub-optimal dietary patterns were observed in this child sample from Western Sydney. Few children were exclusively breastfed to 6 months of age, and most received solids before 6 months. The median diet diversity score was five out of out, indicating moderate diet diversity within the sample. Fibre intakes were low compared to other nutrients, and most children exceeded the intake limits for protein, total fat, saturated fat, and sodium. The thematic analysis revealed mothers’ perceptions of their children’s diet, maternal upbringing, and external advice to influence mothers’ feeding choices. These findings emphasize the urgency of implementing community-based initiatives and support to empower parents with accurate information and culturally relevant guidance, fostering healthier feeding practices, and improving the overall nutritional outcomes for children in Western Sydney.

## Figures and Tables

**Table 1:** Characteristics of the study participants.

	Mothers (*n* = 29)	Children (*n* = 29)
Age (years)	35 ± 3.6 ^1^	2 (1–3)
Sex (*n* (%))		
Female	29 (100.0)	15 (51.7)
Male	0 (0.0)	14 (48.3)
Ethnicity (*n* (%))		
European	15 (51.7)	-
Aboriginal and/or Torres Strait Islander	1 (3.4)	2 (6.9)
Vietnamese	6 (20.7)	-
Chinese	4 (13.8)	-
Indonesian	1 (3.4)	-
Mixed Asian	2 (6.9)	-
Education (*n* (%))		
Certificate or lower	4 (13.8)	-
Diploma, degree, or higher	25 (86.2)	-
In paid employment (*n* (%))	17 (58.6)	-
Socioeconomic status ^2^ (*n* (%))		
IRSAD deciles 1–4	11 (37.9)	11 (37.9)
IRSAD deciles 5–7	6 (20.7)	6 (20.7)
IRSAD deciles 8–10	12 (41.4)	12 (41.4)
Anthropometry		
Weight (kg)	63 (46–105)	12.5 (11–14.8)
Length/height (m)	1.64 ± 0.1	0.9 ± 0.1 ^3^
BMI (kg/m^2^)	23.4 (19.3–40)	16.2 ± 3.0 ^3^
BMI *z*-score	-	0.7 (−1.4–1.5) ^3^

IRSAD: index of relative socio-economic advantage and disadvantage; BMI: body mass index. -: not applicable. ^1^ Data unavailable for *n* = 2 mothers. ^2^ Higher IRSAD deciles correspond to greater socioeconomic advantage. ^3^ Data unavailable for *n* = 7 children.

**Table 2:** Infant feeding history.

	*n* (%)
First feed	
Breastmilk	27 (93.1)
Infant formula	2 (6.9)
Breastfeeding	
Continuing	6 (20.7)
Ceased	23 (79.3)
Ceased < 6 months	4 (17.4)
Ceased 6–12 months	8 (34.8)
Ceased > 12 months	11 (47.8)
Age at introduction to solid foods	
4 months	8 (27.6)
5 months	10 (34.5)
6 months	11 (37.9)
Previously used squeeze pouches ^1^	24 (82.8)
Introduced at <6 months	4 (16.7)
Introduced at 6 months	9 (37.5)
Introduced at >6 months	10 (41.7)

^1^ Age at introduction missing for *n* = 1 participant.

**Table 3:** Energy and nutrient intakes and nutrient adequacy of child sample (*n* = 29).

	Intake Mean ± SD or Median (IQR)	Meeting Requirements ^1^ *n* (%)
Energy (kJ)	6000.7 ± 3093.3	22 (75.9)
Macronutrients		
Protein (g)	56.6 ± 28.0	28 (96.6)
Total fat (g)	44.4 (32.0–72.5)	7 (24.1) ^2^
Monounsaturated fat (g)	16.5 (10.1–24.8)	-
Polyunsaturated fat (g)	5.2 (3.3–8.5)	-
Saturated fat (g)	19.4 (13.5–30.9)	-
Carbohydrates (g)	158.8 (111.9–207.9)	9 (31.0) ^3^
Fibre (g)	17.0 ± 8.0	14 (53.8) ^4^
Micronutrients		
Calcium (mg)	701.0 ± 318.8	23 (79.3)
Iron (mg)	6.3 (4.0–11.6)	23 (79.3)
Sodium (mg)	1516.8 (1077.4–2095.0)	28 (96.6)
Zinc (mg)	6.9 ± 3.4	28 (96.6)
Thiamine (mg)	1.1 (0.6–1.7)	26 (89.7)
Riboflavin (mg)	1.6 ± 0.9	26 (89.7)
Niacin (mg)	12.3 (7.1–17.5)	25 (86.2)
Folate (µg)	284.7 (220.2–408.7)	27 (93.1)
Vitamin B6 (mg)	0.8 (0.6–1.1)	26 (89.7)
Vitamin B12 (µg)	3.0 ± 1.8	27 (93.1)
Vitamin C (mg)	38.6 (28.0–75.2)	22 (75.9)
Vitamin E (mg)	5.2 (3.7–8.2)	16 (55.2)
Other		
Total sugars (g)	68.1 (41.9–89.8)	-
Caffeine (mg)	0.0 (0.0–0.6)	-
Cholesterol (mg)	128.3 (67.5–362.1)	-

-: not applicable. ^1^ Estimated energy requirement (EER) for energy; estimated average requirement, adequate intake, or acceptable macronutrient distribution range (AMDR) for macro- and micro-nutrients. ^2^ Adequate intake for children ≤ 12 months, AMDR (20–35% of EER) for older children. ^3^ Adequate intake for children ≤ 12 months, AMDR (45–65% of EER) for older children. ^4^ No requirements for children ≤ 12 months, so *n* (%) out of 26 children.

**Table 4:** Illustrative quotations regarding mothers’ perceptions of their children’s diet.

Sub-Theme	Quote
Perceptions of a child’s readiness to eat solids	*“She just hit a point where she just started picking up foods off my plate and giving them to herself” (Mother, child aged 9 months)*
Perceptions about a child’s diet	*“Now, she knows exactly what’s in the fridge and she’s always snacking. And it’s quite healthy as well. So, she eats a lot of apples and a lot of fruit.” (Mother, child aged 5 years)*

**Table 5:** Illustrative quotations regarding mothers’ external influences on their feeding decisions.

Sub-Theme	Quote
Advice from family and friends	*“Oh, mum and friends. Friends are because—a couple of mummy friends would because they were just like,” Oh, “they post pictures of their child to say they eat this or broccoli or this at this age…” “So it’s also set further examples and what works for them and tested by them. My mum is because she feeds me, so I trust her with that one as well.” (Mother, child aged 3 years).*
Advice from mother’s groups	*“A lot of the support and the advice I got was from other women in my mother’s group who had done all the research into introducing solids…” (Mother, child aged 18 months).*
Advice from healthcare providers	*“It’s only when we experience something like we’re worried about if he’s not gaining weight well or he’s getting sick, then we would chat to the doctor and nurse about that, but so far, we haven’t really talked much about solid foods.” (Mother, child aged 13 months).*
Information from the internet and social media	*“I did read a lot of books. So I read a lot on weaning books, especially Gina Ford’s book, which talks about when to start baby on solids, what to feed them and everything.” (Mother, child aged 13 months).* *“So I use Facebook forums and groups, and I just look at the information and resources that they have and then just go from it to Google on a theme.” (Mother, child aged 20 months).*
Uncertainty about advice	*“I did a lot of research online and through books and just speaking with other people and came to what was the most common feature. So, a lot of them said berries—they could have a reaction. So I steer clear of them for a little while. Same with honey, it was quite—although there was all different data, there was a majority. So I went with the majority.” (Mother, child aged 2 years).*

**Table 6:** Illustrative quotations regarding mothers’ personal experience and feeding decisions.

Sub-Theme	Quote
Mother’s upbringing and own experiences	*“I guess growing up in an Asian household, rice is number one. So I tend to feed him a lot of rice and noodles.” (Mother, child aged 2 years).* *“Two out of three of my oldest kids are lactose-intolerant. And just the fact that she was quite small … made me think that maybe she had an issue with lactose.” (Mother, child aged 22 months).*

**Table 7:** Illustrative quotations regarding feeding choices and behaviours.

Sub-Theme	Quote
Mother’s choice of foods to provide	*“He’ll eat whatever the family is eating which is a wide range of meats, pasta, rice, potato, salads, veggies, cereal, bread, sandwiches.” (Mother, child aged 2 years).*
Pressure to eat	*“…I always had a thing that he has to finish what I gave him ‘cause, otherwise, he would be behind someone exactly the same age as him.” (Mother, child aged 3 years).* *“So he can eat as much as he likes, but he tells me he’s full, that’s fine, he doesn’t have to finish it but he then can’t have something else…” (Mother, child aged 4 years).*
Giving their child a choice of foods	*“I think maybe you can provide him with options but you still need to watch what you give him.” (Mother, child aged 10 months).* *“…if my child doesn’t wanna finish their meal, I don’t make them. Learning to listen to their bodies and they can come back and finish it later.” (Mother, child aged 5 years).*

## Data Availability

Datasets generated and analysed in this study are not publicly available due to ongoing data analyses, but they will be made available upon reasonable written request to the corresponding author.
